# Spatiotemporal and meteorological relationships in dengue transmission in the Dominican Republic, 2015–2019

**DOI:** 10.1186/s41182-023-00517-9

**Published:** 2023-06-02

**Authors:** Michael A. Robert, Helena Sofia Rodrigues, Demian Herrera, Juan de Mata Donado Campos, Fernando Morilla, Javier Del Águila Mejía, María Elena Guardado, Ronald Skewes, Manuel Colomé-Hidalgo

**Affiliations:** 1 Department of Mathematics and Center for Emerging, Zoonotic, and Arthropod-Borne Pathogens (CeZAP), Virginia Polytechnic Institute and State University, Blacksburg, VA USA; 2 Escola Superior de Ciências Empresariais, Instituto Politécnico de Viana do Castelo, Valença, Portugal; 3 Centro de Investigação e Desenvolvimento em Matemática e Aplicações, Universidade de Aveiro, Aveiro, Portugal; 4 Centro de Investigación en Salud Dr. Hugo Mendoza, Hospital Pediátrico Dr. Hugo Mendoza, Santo Domingo, Dominican Republic; 5 Departamento de Medicina Preventiva y Salud Pública y Microbiología, Facultad de Medicina, Universidad Autónoma de Madrid, Madrid, Spain; 6 Instituto de Investigación Sanitaria del Hospital Universitario La Paz (IdiPAZ), Universidad Autónoma de Madrid, Madrid, Spain; 7 Consorcio de Investigación Biomédica en Red de Epidemiología y Salud Pública (CIBERESP), Instituto de Salud Carlos III, Calle Monforte de Lemos 3-5, 28029 Madrid, Spain; 8 Departamento de Informática y Automática, Escuela Técnica Superior de Ingeniería Informática, Universidad Nacional de Educación a Distancia, Madrid, Spain; 9 Instituto Tecnológico de Santo Domingo (INTEC), Santo Domingo, Dominican Republic; 10 Dirección General de Epidemiología, Ministerio de Salud, Santo Domingo, Dominican Republic

**Keywords:** Dominican Republic, Dengue, Spatial analysis, Correlation lags

## Abstract

Dengue has broadened its global distribution substantially in the past two decades, and many endemic areas are experiencing increases in incidence. The Dominican Republic recently experienced its two largest outbreaks to date with 16,836 reported cases in 2015 and 20,123 reported cases in 2019. With continued increases in dengue transmission, developing tools to better prepare healthcare systems and mosquito control agencies is of critical importance. Before such tools can be developed, however, we must first better understand potential drivers of dengue transmission. To that end, we focus in this paper on determining relationships between climate variables and dengue transmission with an emphasis on eight provinces and the capital city of the Dominican Republic in the period 2015–2019. We present summary statistics for dengue cases, temperature, precipitation, and relative humidity in this period, and we conduct an analysis of correlated lags between climate variables and dengue cases as well as correlated lags among dengue cases in each of the nine locations. We find that the southwestern province of Barahona had the largest dengue incidence in both 2015 and 2019. Among all climate variables considered, lags between relative humidity variables and dengue cases were the most frequently correlated. We found that most locations had significant correlations with cases in other locations at lags of zero weeks. These results can be used to improve predictive models of dengue transmission in the country.

**Supplementary Information:**

The online version contains supplementary material available at 10.1186/s41182-023-00517-9.

## Introduction

Global incidence of dengue fever has increased substantially in recent decades, with the range of dengue expanding from only nine countries before 1970 to at least 129 countries today [1–4]. In addition to its rapid global spread, dengue outbreaks in endemic regions are resulting in increasingly larger numbers of cases and contributing to a growing burden on public health systems. Today, it is estimated that over 390 million people are at risk of contracting dengue [5]. Dengue is primarily distributed across regions of the world with tropical and subtropical climates, although in the past two decades, dengue cases have occurred with greater frequency in temperate zones as well [6–9]. In 2021, 1,254,648 cases and 436 deaths were reported in the Americas [10]. According to the Pan American Health Organization (PAHO) the countries in the Caribbean reporting the most cases of dengue between 2014 and 2021 are the Dominican Republic, Martinique, Guadeloupe, French Guiana, and Cuba, with the Dominican Republic having 60% more cases in that time as the country reporting the second highest number [10]. The Dominican Republic also reports the highest number of severe dengue cases and deaths in the Caribbean [10]. In 2019, the Dominican Republic experienced its largest outbreak to date with a 1145% increase in cases from 2018 [11]. The cumulative incidence in 2019 was 194.85 cases per 100,000 people, which is a 142% increase from the average incidence between 2005 and 2014 [12, 13].

With outbreaks becoming increasingly severe in the Dominican Republic and other regions, it is more imperative than ever to understand drivers of epidemic dengue. Potential drivers of global spread of dengue include increases in urbanization, more frequent global travel, and changes in temperature and precipitation [14–16]. At local scales, transmission of dengue can also be a function of socioeconomic and demographic characteristics, connectivity to other regions, human behavior, volume of tourism, and rates of migration [14–18]. Many of these variables play an important role in developing and sustaining an environment that is suitable for the vectors of dengue, *Aedes aegypti* and *Aedes albopictus*, which in turn amplifies risk of transmission [19–21].

Because there is an inherent delay between human cases of dengue resulting from the intermediate mosquito host and a serial interval of 15–17 days, early detection of new dengue outbreaks can be complicated [22, 23]. In the last decade, efforts have been made to improve early detection of dengue outbreaks by improving surveillance and warning [22, 24–26]. These early warning systems are mathematical and statistical models that integrate data to provide predictions for changes in dengue transmission that may indicate outbreaks. Chief among these data are climate variables such as temperature, precipitation, or humidity which are all positively correlated with *Aedes* mosquito populations and dengue transmission [22]. However, before such early warning systems can be developed, relationships between climate variables and dengue cases must be explored to determine which climate variables are most important to local and regional dengue transmission. Recently, Freitas et al. thoroughly analyzed the 2019 dengue outbreak in the Dominican Republic and found that a model incorporating temperature and rainfall with delays of 2–5 weeks provided good predictions of dengue transmission [27]. This work marks an important first step in developing better predictive models for the country.

Herein, we aim to build upon this work by analyzing dengue activity in the Dominican Republic between 2015 and 2019 and exploring relationships between climate variables and dengue cases. We present descriptive analysis of each data set used in the study along with analysis of correlations in lags between variables. We further investigate correlations in lags between provinces in the Dominican Republic to understand potential movement of dengue throughout the country. The work presented herein provides a foundation on which statistical and mathematical models can be constructed to further study drivers of previous outbreaks and to predict future outbreaks.

## Materials and methods

### Study site

This study was conducted in the Dominican Republic, a Caribbean country that occupies the eastern two-thirds of the Island of Hispaniola. The 2019 estimate of the population size of the Dominican Republic is 10,448,499 people [27]. The country is divided geopolitically into 31 provinces and Distrito Nacional (the capital city) [28].

The Dominican Republic has perhaps the most diverse climate of all of the Caribbean islands because of the presence of high mountains and abundant coastal regions [29]. Much of the country, however, has a tropical climate with mean annual temperatures ranging between 22 and 31 °C [29–32]. Rainy seasons vary geographically with the northern part of the country experiencing heavier rain from November to January and much of the rest of the country having its rainy season May–November. Mean annual precipitation in the country ranges between 400 mm in the southwest to more than 2200 mm in the mountain regions [29]. The majority of southern coastal regions experience a mean rainfall of around 1000 mm while northern coasts typically have a higher mean annual rainfall of 1600 mm or more [29].

**Fig. 1:**
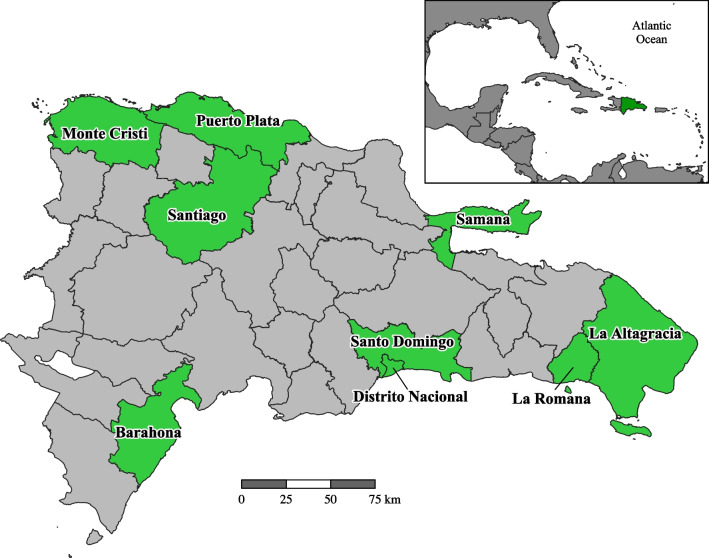
Provinces of the Dominican Republic. Provinces at the focus of this study are highlighted in green

In this work, we focus on nine provinces throughout the country. These nine were chosen because they are the provinces for which we were able to obtain both meteorological and epidemiological data between 2015 and 2019. The nine provinces included in this study are Barahona, La Altagracia, La Romana, Monte Cristi, Puerto Plata, Samaná, Santiago, Santo Domingo, and Distrito Nacional (Fig. 1). The provinces cover each of the three major regions of the country: North (Monte Cristi, Puerto Plata, Santiago, Samaná), South (Barahona), and East/Southeast (Santo Domingo, Distrito Nacional, La Romana, La Altagracia). The nine provinces include 6,295,775 people (2019 estimate), representing 60.78% of the total population of the country.

### Data collection

#### Dengue cases

**Fig. 2:**
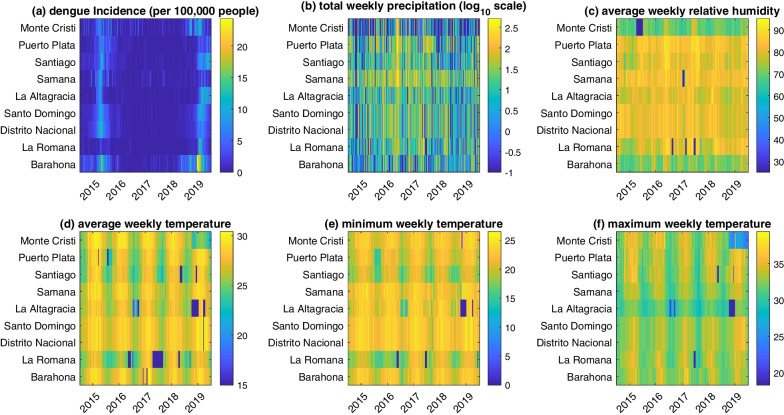
Time series of epidemiological and meteorological metrics across the five years and nine provinces at the focus of this study. Provinces are arranged by latitude (northernmost to southernmost). **a** Dengue incidence per 100,000 people; **b** total weekly precipitation (mm^3^; log scale); **c** average weekly relative humidity (%); (**d**–**f**) average, minimum, and maximum weekly temperature, respectively (°C)

The number of weekly reported cases for the period January 2015 to December 2019 was provided by Sistema Nacional de Vigilancia Epidemiológica de la Dirección General de Epidemiológica (Ministerio de Salud Pública). The epidemiological week was defined as Sunday to Saturday. Cases include suspected and laboratory-confirmed cases aggregated at the province level according to surveillance definitions [33]. Dengue Incidence Rate (DIR) was calculated using the number of new cases, divided by the local population each year, multiplied by 100,000 inhabitants. Figure 2a shows dengue incidence for the five years across the nine provinces included in the study.

#### Meteorological data

Meteorological data were obtained by supplementing official national data (from ONAMET) with data provided by the U.S. National Aeronautics and Space Administration (NASA). Previous studies have confirmed this approach for collecting data, especially where there are gaps in reliable data [24, 34]. We calculated summary values (minimum, maximum, mean, sum) of meteorological parameters by epidemiologic weeks. Figure 2b–d show average weekly temperature, total weekly precipitation, and average weekly relative humidity for the 5 years across the nine provinces included in the study.

#### Population data

We obtained population data from Oficina Nacional de Estadísticas (ONE) [27]. This data includes the total population and the population density for each province (Table 1). Distrito Nacional has the highest population density, and Santo Domingo province has the highest population.

**Table 1:** Demographic characteristics from provinces of the Dominican Republic included in this study, 2019

Province	Population size	Population density (individuals/km^2^)
Barahona	189,149	1160.28
La Altagracia	345,822	114.88
La Romana	270,166	1456.26
Monte Cristi	116,605	255.99
Puerto Plata	332,386	653.01
Samaná	111,217	130.27
Santiago	1,038,044	369.90
Santo Domingo	2,855,892	1059.03
Distrito Nacional	1,036,494	9924.30

Data was obtained from ONE [27]

### Statistical analysis

#### Summary statistics

We conducted a preliminary statistical analysis of epidemiological data to highlight changes in dengue cases across provinces and across years. We calculated summary statistics of dengue cases for the entire country each year. We focus much of our analysis of cases on the years 2015 and 2019, when large epidemics took place. Our initial findings support subsequent analysis at the province level to assess the association between explanatory variables and the distribution of the disease over space and time. We calculate dengue incidence in each province in 2015 and 2019 as well as descriptive statistics such as mean, maximum, minimum, and standard deviation of meteorological variables for each province. The results were obtained by calculations in Microsoft Excel, through the Excel Data Analysis Tool. Maps with spatial data were generated by QGIS 3.16.6. These maps improve our understanding of dengue transmission in different regions and how transmission evolves spatially over time [35].

##### Correlated lags analysis

We compared time series of dengue case data across provinces with climate data by conducting an analysis of correlations of lags between data sets. We assume a unidirectional relationship between dengue cases and meteorological variables. We first implemented a standard prewhitening approach to remove any effects of autocorrelation within data sets [36]. We fit each time series data set to seasonal autoregressive integrated moving average (SARIMA) models. We calculated cross-correlation functions between residuals of time series for different weekly summary data for these variables with lags up to 10 weeks. We chose this cutoff for lags because of the relatively short time scale of our data (5 years) and because it is a biologically reasonable time for weather events to impact mosquito development and disease transmission given the development time and generation time of mosquitoes as well as the serial interval of dengue [23, 37, 38]. We tested for significance of correlated lags with a two-tailed t-test to test the null hypothesis that the correlation was equivalent to 0. We report the lags with the highest correlation along with p-values at the 0.10, 0.05, and 0.01 confidence levels.

We also conducted a correlated lag analysis among provinces. We calculated correlations between lags in residuals of time series of cases in each province. We determined the significance of these correlations with a two-tailed t-test to test the null hypothesis that the correlation was equivalent to 0. We report the lags with the highest correlation along with p-values at the 0.10, 0.05, and 0.01 confidence levels. All correlation analyses were conducted in R 4.1.0 [39]. Fitting of SARIMA models was conducted using the auto.arima() function in the forecast package [40, 41]. Cross-correlation functions were calculated with the ccf() function in the R base installation [42].

## Results

### Dengue cases: spatiotemporal analysis

We first calculated descriptive statistics for dengue cases in the country each year (total cases, mean cases per week, standard deviation of cases per week, minimum number of cases per week, maximum number of cases per week, and the dengue incidence rate per year). Table 2 presents these results. In 2015 and 2019 there were major outbreaks with dengue incidence rates of 168.69 and 194.27 cases per 100,000 residents, respectively.

**Table 2:** Descriptive statistics for dengue cases each year, 2015–2020

	2015	2016	2017	2018	2019	2020
Total cases	16,836	6559	1335	1538	20,123	3070
Mean (per week)	323.77	126.13	25.67	29.58	386.98	59.04
St. Dev (per week)	40.64	15.62	1.44	2.47	37.49	15.50
Min. (per week)	66	15	5	5	57	0
Max. (per week)	1140	530	57	87	888	417
DIR (per 100,000 people)	168.69	65.10	13.13	14.98	194.27	29.38

**Fig. 3:**
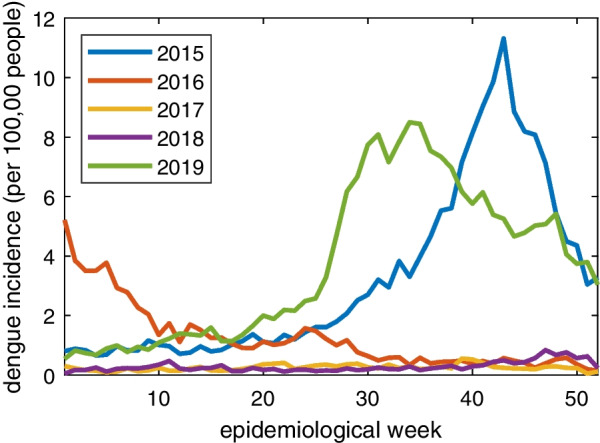
National dengue incidence by week from 2015 to 2019 in the Dominican Republic

**Fig. 4:**
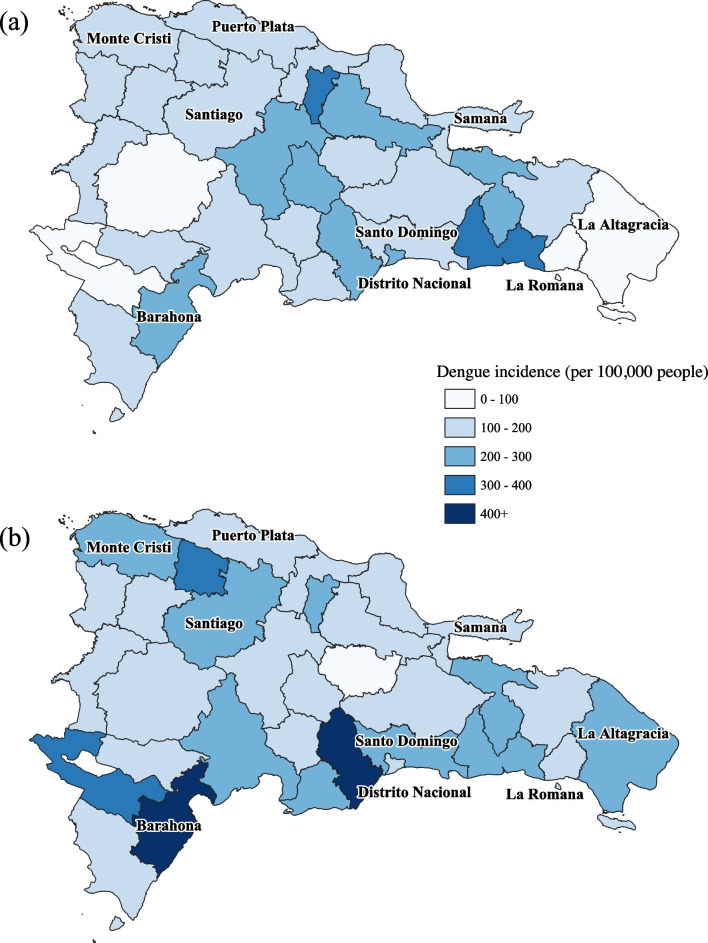
Spatial description of dengue incidence in 2015 (**a**) and 2019 (**b**)

The two largest outbreaks each started during late spring (April–May) and continued for approximately one year (Fig. 3). The 2015 outbreak peaked in October, while the 2019 outbreak peaked in August. Central provinces experienced the highest incidence during the 2015 outbreaks, while in 2019, provinces in the north and south experienced the highest incidence (Fig. 4). The increase in incidence in the northern and southern provinces could be related to socioeconomic factors or differences in climate variables between the 2 years. Barahona in the southwest, along with Hermanas Mirabel, Sánchez Ramirez, and San José de Ocoa in the center and Hato Mayor in the east all experienced similarly high incidence in both 2015 and 2019. Puerto Plata in the north along with Distrito Nacional in the southeast and many other coastal provinces experienced similarly lower incidence in both outbreaks. Puerto Plata and Distrito Nacional are both popular destinations for international travel and thus are likely to employ more aggressive mosquito control and dengue prevention practices. Table 3 shows incidence calculations for the nine provinces of focus for this study along with the national incidence for both 2015 and 2019.

**Table 3:** Dengue incidence per 100,000 people by province for the 2015 and 2019 outbreaks

Year	Barahona	La Altagracia	La Romana	Monte Cristi	Puerto Plata	Samaná	Santiago	Santo Domingo	Distrito Nacional	Dominican Republic
2015	273.9	87.6	30.7	132.1	114.3	131.3	148.5	162.0	197.6	168.7
2019	456.2	206.6	153.0	250.8	104.7	72.2	216.1	210.7	163.9	194.3

### Climate variables and dengue cases

The majority of the Dominican Republic has a tropical climate with hot temperatures all year and the warmest months being May to October. There is a rainy season between late April and October, while the northern coast, exposed to the trade winds, is rainy throughout the year. On the southern coast, there is a considerable amount of precipitation because it is not protected by mountains. As a Caribbean country, the rains occur mainly as short showers and thunderstorms which are sometimes intense and often concentrated in short periods of time. Table 4 summarizes the climate statistics for the nine provinces studied in the two outbreaks. In both years, the values for all climate variables did not vary so much.

**Table 4:** Summary statistics of climate variables

	2015					2019				
	Min. temp	Mean temp	Max. temp	Total precip	Mean RH	Min. temp	Mean temp	Max. temp	Total precip	Mean RH
Barahona	21.6 (19–25.5)	27.6 (24.5–30)	33.0 (30.4–38)	9.7 (0–79.5)	69.9 (63.3–81.2)	21.7 (18–25)	27.4 (24.9–29.9)	32.8 (30.6–35.8)	15.0 (0–99.6)	72.5 (64.2–83.1)
La Altagracia	22.0 (17.1–26.2)	27.3 (25.2–29.4)	31.4 (29.6–33.4)	18.2 (0–272.6)	78.4 (64.8–84.2)	21.3 (17–24)	26.8 (24.9–28.9)	31.2 (28.8–33)	12.0 (0–61.4)	77.2 (71.0–83.2)
La Romana	19.0 (15.5–22)	26.3 (24.0–28.6)	33.3 (31–36)	13.0 (0–130.5)	76.2 (62.3–84.2)	19.4 (14.4–23.2)	26.3 (22.9–28.5)	33.0 (30.5–35.8)	21.4 (0–150.1)	82.7 (76.3–90.2)
Monte Cristi	21.7 (18.5–24.9)	27.8 (23.8–30.3)	33.8 (30.8–37.4)	10.3 (0–165.5)	68.5 (63.2–81.0)	21.4 (2.2–25)	24.5 (20.3–26.9)	27.3 (23–35.9)	7.6 (0–107.5)	69.3 (60.6–77.6)
Puerto Plata	20.8 (15.8–23.7)	27.0 (23.6–29.7)	33.5 (30–37.4)	24.0 (0–225.9)	81.4 (68.3–89.0)	20.9 (15.6–23.6)	27.2 (24.2–29.9)	33.8 (30.2–37.7)	17.6 (0–149)	81.8 (77.4–90.5)
Samaná	22.7 (29.5–35)	27.7 (25.3–30.2)	32.4 (29.5–35)	39.4 (0–201.9)	83.6 (71.2–91.8)	22.1 (14–25)	27.6 (24.7–29.8)	32.8 (29.5–35)	30.7 (0–148)	83.2 (77.0–89.4)
Santiago	19.6 (15–22.8)	26.6 (23.5–29.5)	33.3 (29.7–37.7)	13.5 (0–50.2)	78.3 (65.1–88.0)	19.2 (15–22)	26.7 (23.4–29.3)	33.8 (30.2–37.4)	16.9 (0–140.2)	79.8 (69.7–88.8)
Santo Domingo	23.0 (20.8–26)	27.9 (26.0–29.9)	32.9 (31–37.2)	22.86 (0–126.6)	82.5 (72.0–91.5)	22.9 (20.1–26)	28.3 (26.1–30.4)	33.8 (31–37)	13.7 (0–83.8)	78.6 (72.5–84.1)
Distrito Nacional	23.0 (20.8–26)	27.9 (26.0–29.9)	32.9 (31–37.2)	22.86 (0–126.6)	82.5 (72.0–91.5)	22.9 (20.1–26)	28.3 (26.1–30.4)	33.8 (31–37)	13.7 (0–83.8)	78.6 (72.5–84.1)

Statistics are calculated per week. Averages across the year are shown above ranges (Min.–Max.) of each variable in parentheses below. All temperatures are given in (°C), and precipitation is given in mm^3^. Relative humidity (RH) values are measured in percentages

**Fig. 5:**
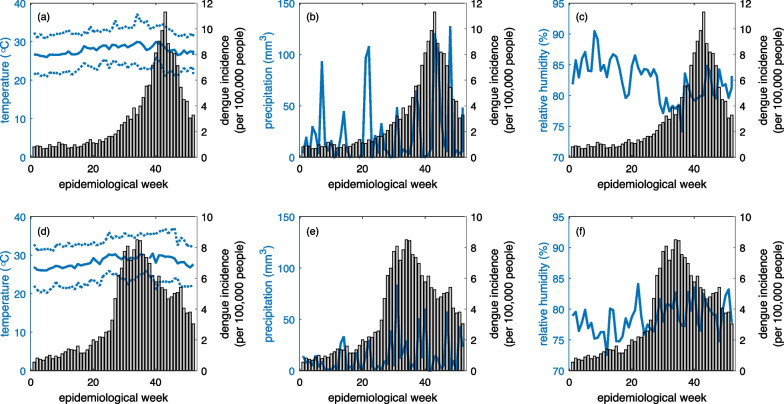
Climate variables and dengue cases in 2015 (**a**–**c**) and 2019 (**d**–**f**). Variables included are (**a**, **d**) temperature (mean temperature is given by the solid curve; °C); (**b**, **e**) precipitation (mm^3^); and (**c**, **f**) relative humidity (%)

Although average values and ranges of climate variables are useful for pointing out variations across years, it is important to consider the temporal variation in climate variables and how they might relate to dengue outbreaks. As an example, we show in Fig. 5 temporal variation in climate variables and dengue cases in 2015 and 2019 in Distrito Nacional. Behavior across provinces were similar and are excluded here for brevity. Temperature and relative humidity are relatively stable throughout the year, although temperatures increase from the beginning of each year until epidemiological weeks 30–35. The cumulative precipitation per week is rather variable and could potentially have more influence on dengue cases. We investigate this relationship, along with relationships between weekly variation in other climate variables and dengue transmission, in the next section.

### Cross-correlation analysis

#### Correlations in lags between dengue cases and climate variables

Table 5 contains correlations between lags in climate variables and dengue cases in the 9 provinces. Although we tested 14 variables, we present only 9 here. Additional file 1: Table S1 in supporting information shows values for the remaining variables we tested. Maximum and average weekly relative humidity were the variables most often significantly correlated with dengue cases at the α = 0.05 or stronger confidence level (6 and 5 of 9 provinces, respectively), followed by weekly minimum temperature (4 of 9 provinces), relative humidity range, mean daily temperature, and maximum weekly temperature (3 of 9 provinces).

**Table 5:** Correlations in lags between dengue cases and climate variables

Province	Mean daily temperature	Mean daily temperature range	Max. weekly temperature	Min. weekly temperature	Mean daily precipitation	Total weekly precipitation	Mean daily RH	Max. weekly RH	Mean weekly RH range
Barahona	− 2 (0.1020)	− 3 (− 0.0985)	− 2 (0.0900)	− 6 (0.1451)**	− 8 (0.0597)	− 8 (0.0561)	− 4 (− 0.1589)**	− 4 (− 0.1536)**	− 5 (0.1452)**
La Altagracia	− 2 (0.0512)	− 2 (− 0.0941)	− 3 (0.0394)	− 2 (0.0631)	− 6 (0.0924)	− 6 (0.0968)	− 6 (0.1344)**	− 10 (− 0.1626)***	− 1 (− 0.0894)
La Romana	− 8 (0.0729)	− 1 (0.1483)**	− 2 (− 0.1279)**	− 2 (0.0843)	− 9 (− 0.0824)	− 9 (− 0.0832)	− 9 (− 0.1454)**	− 4 (− 0.1098)*	− 9 (0.1873)***
Monte Cristi	− 9 (− 0.0589)	− 8 (0.0619)	− 4 (0.1739)***	− 6 (0.1393)**	− 3 (− 0.0858)	− 3 (− 0.0878)	− 9 (0.1394)**	− 3 (− 0.1379)**	− 5 (− 0.1197)*
Puerto Plata	− 5 (− 0.1450)**	− 1 (0.0783)	− 5 (− 0.1495)**	− 1 (− 0.1102)*	− 2 (− 0.0664)	− 2 (− 0.0663)	− 10 (− 0.1594)**	− 2 (− 0.1013)	− 10 (0.2372)***
Samaná	− 6 (0.0909)	− 1 (0.1549)**	− 8 (0.0997)	− 7 (0.1241)**	0 (− 0.0927)	0 (0.0953)	0 (− 0.0906)	− 4 (− 0.0650)	− 1 (− 0.0625)
Santiago	0 (− 0.0946)	− 6 (0.1023)*	− 2 (0.1055)*	− 5 (0.0722)	0 (0.1072)*	0 (− 0.1070)*	− 2 (0.0739)	− 5 (0.1104)*	− 5 (0.1108)*
Santo Domingo	− 1 (0.1568)**	0 (0.0779)	− 1 (0.1175)*	− 1 (0.1506)**	− 3 (− 0.0876)	− 3 (− 0.0942)	− 10 ( 0.1669)***	− 10 (− 0.1888)***	− 4 (− 0.0719)
Distrito Nacional	− 1 (− 0.1351)**	− 1 (− 0.0955)	− 4 (0.1004)	− 9 (0.1074)*	− 7 (− 0.0889)	− 7 (− 0.0850)	− 3 (0.1150)*	− 3 (0.1256)**	− 3 (0.0810)

Lags are given as the number of weeks prior to dengue cases. Lags are listed with correlations in parentheses. Stars indicate confidence levels for testing significance: ***p < 0.01, **p < 0.05, *p < 0.10

In Barahona, La Altagracia, La Romana, Monte Cristi, Puerto Plata, and Santo Domingo, lags between dengue cases and average relative humidity were significantly correlated at the α = 0.05 or stronger confidence level with lags of 4–10 weeks. For most provinces, these correlations were negative, suggesting that, for example, decreasing average relative humidity may be associated with increases in dengue cases. Lags of 3–10 weeks between dengue cases and maximum relative humidity were significantly correlated at the α = 0.05 or stronger confidence level in Barahona, La Romana, Monte Cristi, Santo Domingo, and Distrito Nacional. For all provinces except Distrito Nacional, these correlations were also negative. For lags with the weekly range of relative humidity, lags of 5–10 weeks were significantly positively correlated at the α = 0.05 or stronger confidence level for Barahona, La Romana, and Puerto Plata provinces, suggesting higher ranges in weekly relatively humidity could be associated with dengue cases. For provinces for which we found significant positive correlations between dengue cases and relative humidity variables, rates of increases in cases lagged behind those of other provinces, suggesting that perhaps timing of cases in provinces relative to one another played a role in these relationships. We explore this further later in this section.

In general, correlations between lags in dengue cases and temperature variables were significant less often than those we found for relative humidity; however, lags between cases and minimum weekly temperature were significantly correlated at the α = 0.05 or stronger level for lags of 1–7 weeks for the provinces of Barahona, Monte Cristi, Samaná, and Santo Domingo. In all four of these provinces, correlations were positive, suggesting that higher minimum weekly temperatures were associated with more dengue cases. Lags of 2–5 weeks between maximum weekly temperature and dengue cases were also strongly correlated at the α = 0.05 or stronger level for La Romana, Monte Cristi, and Puerto Plata provinces, although correlations in La Romana and Puerto Plata were negative whereas the correlation in Monte Cristi was positive. For mean daily temperature, lags of 1–5 weeks between this variable and dengue cases were significantly correlated at the α = 0.05 or stronger confidence level. Notably, lags of 1 week between mean daily temperature and dengue cases were significant in Santo Domingo and Distrito Nacional; however, the direction of these correlations differed. Again, it is possible that these differences in the directions of relationships are a result of lags between the timing of outbreaks in different provinces.

No correlations of lags with total precipitation and average precipitation were significant at the α = 0.05 confidence level. This result is surprising because we would expect dengue transmission in tropical climates to be positively correlated with precipitation given the important role of water in the mosquito’s life cycle [43].

The provinces with the most significantly correlated lags in climate variables at α = 0.05 or stronger confidence level were Santo Domingo (7 variables); Puerto Plata (6); Barahona, La Romana, and Distrito Nacional (5); and Monte Cristi (4). For all other provinces included in the study, only 2 or fewer climate variables were significantly correlated.

#### Correlations in lags between cases in different provinces

Almost all of the largest correlations in lags between cases in different provinces were significant (p < 0.05), and most were highly significant (p < 0.01). Only three of 72 tested correlations were not significant at the α = 0.05 or stronger confidence level: cases in Barahona as a predictor of cases in Distrito Nacional, La Altagracia as a predictor for Santiago, and Santo Domingo as a predictor for Santiago. In most cases, correlations between provinces with a lag of τ = 0 were the strongest (20 of 72 or 27.78%), suggesting that cases were largely synced across provinces (Table 6). Correlations with lags of 2 weeks (16.67%), 1 week (12.5%), and 4 weeks (11.11%) were also among some of the strongest, indicating that cases across the country closely followed cases in other parts of the country.

**Table 6:** Correlations in lags (weeks) between dengue cases in each province

Province	Barahona	La Altagracia	La Romana	Monte Cristi	Puerto Plata	Samaná	Santiago	Santo Domingo	Distrito Nacional
Barahona		− 9 (− 0.2041)***	− 2 (0.2054)***	− 9 (− 0.2078)***	− 2 (0.2620)***	− 2 (0.3217)***	− 2 (0.1606)***	− 9 (− 0.1405)**	− 6 (− 0.1457)**
La Altagracia	− 1 (0.2081)***		− 6 (0.1777)***	− 4 (0.2503)***	0 (0.1770)***	0 (0.2164)***	− 1 (0.1786)***	0 (0.2372)***	− 7 (0.1373)**
La Romana	− 6 (0.1652)***	− 1 (0.1689)***		− 4 (0.1910)***	− 8 (0.2741)***	− 5 (− 0.3330)***	− 1 (0.2104)***	− 4 (0.1881)***	− 4 (0.2411)***
Monte Cristi	− 2 (0.1857)***	− 2 (0.2484)***	− 2 (0.1886)***		− 2 (0.1759)***	− 4 (0.2388)***	0 (0.2697)***	0 (0.1716)***	− 5 (0.1742)***
Puerto Plata	0 (0.1484)**	− 1 (0.2192)***	0 (0.2200)***	− 1 (0.2295)***		0 (0.4782)***	− 1 (0.2863)***	0 (0.2653)***	− 6 (0.1401)**
Samaná	− 10 (0.1281)**	0 (0.2164)***	0 (0.3139)***	− 2 (0.2470)***	0 (0.4782)***		− 1 (0.4216)***	0 (0.1274)**	− 9 (− 0.1911)***
Santiago	0 (0.1421)**	− 2 (0.0660)	0 (0.1405)**	0 (0.2697)***	0 (0.1419)**	− 2 (0.1582)**		0 (− 0.0975)	− 4 (0.1850)***
Santo Domingo	− 7 (0.1247)**	0 (0.2372)***	− 7 (0.2073)***	0 (0.1716)***	0 (0.2653)***	− 10 (0.1439)**	− 8 (0.1358)**		− 8 (0.2567)***
Distrito Nacional	− 5 (− 0.1079)*	− 3 (0.1837)***	− 2 (0.2474)***	− 4 (0.1809)***	− 3 (0.1894)***	− 2 (0.3399)***	− 4 (0.1973)***	− 1 (− 0.1448)**	

In this table, the provinces along the columns are the predictor variables. Lags are listed with correlations in parentheses. Stars indicate confidence levels for testing significance: ***p < 0.01, **p < 0.05, *p < 0.10

Cases in Puerto Plata and Santo Domingo were generally in sync with cases in other regions, with cases in both provinces with a lag of τ = 0 having strong positive correlations with cases in four of the eight other provinces. In fact, cases in Puerto Plata had strong positive correlations with cases in all other provinces with a lag of 0–3 weeks. The only exception is Samaná (τ = − 8). For Santo Domingo, cases were positively correlated with cases in other provinces with a lag of 0 or 4 weeks, but cases were negatively correlated with cases in Barahona (τ = − 9) and Distrito Nacional (τ = − 1). It is possible that the strong positive correlations with short-term lags between cases in Puerto Plata and Santo Domingo are due either population size, high rates of tourism, or some combination thereof.

Cases in Barahona, La Romana, Samaná, and Santiago also were positively correlated with all other provinces with lags of 0–10, 0–7, 0–10, and 0–8 weeks, respectively. In the remaining provinces, correlations were mostly positive but cases in each of these five provinces were negatively correlated with cases in one other province. These correlations were with cases at lags of 5–9 weeks, indicating that perhaps decreases in cases in some provinces preceded increases elsewhere (or vice-versa) by several weeks. Moreso than in other provinces, cases in Samaná, Monte Cristi, and La Romana were correlated with cases in other provinces with lags greater than one week, indicating that cases in these provinces may often occur 2 or more weeks before cases in other provinces. Notably, cases in Distrito Nacional were correlated with cases in all other provinces with lags of 4–9 weeks, suggesting that increases in transmission in the capital district may precede outbreaks elsewhere in the country.

## Discussion

Herein we characterized dengue incidence at the province level in the Dominican Republic between 2015 and 2019, a period in which the country and the Caribbean region experienced two important epidemics. We focused our study on nine provinces that included all major geographic regions of the country that represented different climate patterns. In our study, we observed different potential drivers of dengue activity in different regions of the country. We anticipate that this study will be a foundation upon which early warning systems and models aimed at predicting dengue activity may be built.

We noted that both major outbreaks (2015 and 2019) occurred after the 30th epidemiological week, which corresponds approximately to late July. This, together with the fluctuations noted even in the years in which no epidemic occurred, indicate a seasonal pattern of dengue transmission. When comparing the epidemiology of dengue in the Dominican Republic with the epidemiology throughout the region of the Americas, a similar behavior was observed for 2015 and 2019, the latter being the year with the highest number of cases recorded in the history of dengue in the Americas [28, 44]. The reduction in the number of cases between 2016 and 2018 could be explained in part by the vector control actions implemented by the Ministry of Health, the adaptation of the pathogen, reduction of susceptible population, or partial immunity to dengue conferred by the wave of Zika virus that moved through the region between 2015 and 2016 [45, 46]. Another possible factor that could have an impact on dengue transmission is the El Niño Southern Oscillation (ENSO). During 2015 and at the beginning of 2019 there were warm periods related to ocean–atmosphere temperature [47]. These results are in line with previous studies that found evidence that ENSO is associated with dengue outbreaks [48, 49]. Considering the number of dengue cases that occurred in 2015 and 2019, the ENSO phenomenon is an important factor to be considered in any early warning system. Even with the short period of time in our analysis (5 years), the major outbreaks corresponded to the only years that were classified as warm periods in relationships to ocean–atmosphere features.

We found that the southwestern province of Barahona had the largest dengue incidence in both 2015 (273.9 per 100,000 people) and 2019 (456.2). Furthermore, dengue activity in Barahona preceded dengue activity in six other provinces by 1–10 weeks, and among all the provinces studied here, cases in Barahona were most often negatively correlated at longer time lags (6–9 weeks) with cases elsewhere, suggesting that outbreaks beginning in Barahona may be starting to die out as outbreaks are still growing in other provinces. It is possible that new cases are introduced to the Dominican Republic in this region through immigration from Haiti or via tourism. It is also possible that individuals who have dengue must travel to other provinces for medical care given that this province has only one public hospital [50]. This could lead to movement of cases into other provinces and throughout the country. Cases in Distrito Nacional preceded cases in all other provinces. Distrito Nacional is the most densely populated among the provinces we considered, and as the capital district, it is important to the national economy and tourism. It is highly connected to other provinces in the country through highways which facilitate movement of people and potentially mosquitoes via movement of tires and other containers that serve as breeding sites [51–53]. This result is consistent with other studies showing the importance of population-dense urban areas in regional and national transmission of dengue [54, 55]. These initial findings from the present work suggest that provinces such as Barahona and Distrito Nacional will be important in the development of future predictive models and early warning systems.

In fact, our analyses of lags in cases between provinces could help determine how cases spread spatially in the country by identifying “source” provinces where dengue cases begin (such as Barahona and Distrito Nacional) and “sink” provinces where dengue cases later appear. For example, cases in La Romana and Santo Domingo often trailed cases in other parts of the country. Both provinces are home to several popular tourist attractions and may benefit from increased surveillance and vector control [56]. It is possible that as cases are reported elsewhere in the country, control efforts delay significant amounts of transmission in these provinces. Despite these lags found, most of the lags between provinces were zero, indicating that outbreaks occurred throughout most of the region at about the same time. This could be explained by human movement inside the country. A similar study in the country with Zika virus also suggested that the human mobility and the infrastructure level of each region could influence the transmission of diseases that had *Aedes* aegypti as a vector [57].

We analyzed climate variables that could contribute to the dengue transmission cycle by their impacts on the *Ae. aegypti* life cycle. When all five years of dengue case and climate data are considered, lags between relative humidity variables and dengue cases were most highly correlated, indicating relative humidity as a good predictor of dengue transmission throughout the region. This is consistent with previous studies showing strong correlations between dengue cases and average relative humidity, whether those correlations are positive [58, 59] or negative [60]. Our results showing different directional relationships between dengue and relative humidity (i.e., positive correlations for some provinces and negative correlations for others) emphasize the importance of considering effects of local climate variables on dengue cases and highlight a need for more thorough data collection and analyses of these relationships.

Lags with temperature variables, too, were significantly correlated with cases in many provinces. This result is supported by work showing that temperature influences dengue transmission through its impacts on both the vector life cycle and the virus [15, 19, 61–63]. Surprisingly, lags between precipitation and dengue cases were not found to be significantly correlated with cases in any of the provinces. In studies of other tropical regions, precipitation and humidity are often found to be positively correlated with arbovirus activity [29, 58, 59]. It is possible that this is because the Dominican Republic’s unique topography interferes with weather patterns and results in having rainy seasons at different times of the year in different regions [29]. The landscape of the Dominican Republic is composed of chains and valleys with active faults which decreases precipitation from northeast to southwest in winter and spring and increases aridity in western areas where fewer mountain chains are observed. The mountain chains serve as a barrier to trade winds, affecting the humidity of such areas. Additionally, during short periods of time in April and in November, there is a subtropical atmosphere which is influenced by anomalies in sea surface temperatures [29]. While cases could be impacted locally by changes in precipitation, this may not correspond to times at which dengue transmission is occurring elsewhere, which may lead to impacts on correlation. These results are in line with the ones achieved in [64], showing that temperature and humidity have impacts on the transmission chain.

## Conclusions

The short period of data included in this study is insufficient for making strong characterizations of relationships. However, in this work we developed a better understanding of which variables have been most strongly associated with dengue cases in this time frame, which includes two important outbreaks. Both outbreaks occurred in the middle of the year, which indicate a need for more heightened vigilance during this period of the year. Additionally, humidity and temperature are the climate variables with the highest correlations with the number of cases. These findings will help inform future work for building predictive models that incorporate climate and spatiotemporal data to characterize province risk and refine public health responses. This initial analysis may also provide the foundation for models based on ARIMA, SARIMA, SARIMAX, or other frameworks for predictive models [65–67]. A reliable warning system built on such models and adapted to the intrinsic characteristics of each province (namely climate, demographics, and landforms) could help in the monitoring both the vector and arbovirus transmission [68]. This in turn could lead to a faster intervention of health and entomological authorities, thus decreasing dengue incidence.

This study contributes an important analysis of recent dengue transmission on which more complex spatiotemporal analyses can be conducted. For instance, Distrito Nacional has the most correlations in dengue cases with lags greater than 0 weeks, and cases in Barahona with longer time lags are highly correlated with other regions. It is possible these areas could provide early warning of nationwide outbreaks. The general characterizations of climate and dengue activity along with the correlated lags analysis across the nine provinces included here provide a foundation upon which future studies may build to investigate more intricate relationships between dengue and climate, human movement, and human activity.

## Supplementary Information

**Additional file 1**: **Table S1**. Correlations in lags between dengue cases and climate variables not included in the main text. Lags are given as the number of weeks prior to dengue cases. Lags are listed with correlations in parentheses. Stars indicate confidence levels for testing significance: *** p<.01, ** p< .05, *p <.10.

## Data Availability

The datasets analyzed during the current study are available https://github.com/marobert-biomath/dominican_republic_dengue.
