# Supplementary material for: Spatiotemporal and meteorological relationships in dengue transmission in the Dominican Republic, 2015–2019
Source: Trop Med Health. 2023 Jun 2;51:32. doi: 10.1186/s41182-023-00517-9 (PMC10236710; doi:10.1186/s41182-023-00517-9)
Supplement: Supplementary file 1 — Additional file 1: Table S1. Correlations in lags between dengue cases and climate variables not included in the main text. Lags are given as the number of weeks prior to dengue cases. Lags are listed with correlations in parentheses. Stars indicate confidence levels for testing significance: *** p<.01, ** p< .05, *p <.10. [file 41182_2023_517_MOESM1_ESM.docx]

| **PROVINCE** | **Maximum Daily Temperature Range** | **Minimum Daily Temperature Range** | **Mean Maximum Daily Temperature** | **Mean Minimum Daily Temperature** | **Minimum Relative Humidity** |
| --- | --- | --- | --- | --- | --- |
| **Barahona** | -10  (-0.0935) | -7  (-0.0672) | -2  (0.0581) | -4  (-0.1082)* | -4  (-0.1791)*** |
| **La Altagracia** | -10  (-0.1069)* | -10  (0.1190)* | -8  (0.0420) | -2  (0.0840) | -6  (0.0958) |
| **La Romana** | -2  (-0.0675) | -2  (-0.0921) | -3  (-0.0891) | -9  (0.1005) | -9  (-0.2264)*** |
| **Monte Cristi** | -4  (-0.0811) | -10  (-0.0681) | -4  (0.0969) | -4  (0.0806) | -4  (-0.1277)** |
| **Puerto Plata** | -2  (0.1619)*** | -1  (0.0757) | -5  (-0.1212)* | -5  (-0.1066)* | -10  (-0.3015)*** |
| **Samaná** | -1  (0.0890) | -1  (0.1154)* | 0  (0.0913) | -6  (0.0824) | 0  (-0.0864) |
| **Santiago** | -5  (-0.1077)* | -8  (0.1030)* | -3  (-0.0838) | -5  (0.1034)* | -7  (-0.0970) |
| **Santo Domingo** | -3  (-0.1328)** | 0  (0.0798) | -1  (0.1238)** | -1  (0.1456)** | -10  (-0.0817) |
| **Distrito Nacional** | -6  (0.1115)* | -5  (-0.1357)** | -3  (-0.1298)** | -1  (-0.1218)** | -5  (-0.0553) |

**Table S1**. Correlations in lags between dengue cases and climate variables not included in the main text. Lags are given as the number of weeks prior to dengue cases. Lags are listed with correlations in parentheses. Stars indicate confidence levels for testing significance: *** p<.01, ** p< .05, *p <.10.
